# Death from the Inhalation of Amylene

**Published:** 1857-07

**Authors:** 


*Death from the Inhalation of Amylene.*—We have noticed in previous numbers of the Journal, the new anæsthetic lately introduced by Dr. Snow, of London. This agent has been highly extolled by many experimenters for its innoxiousness. MM. Debout and Thourdes have declared that it was even safer than sulphuric ether, whilst Mr. Clarke, of Bristol, believes "that it would be impossible to get too much into the system." The unfortunate result of the case recently occurring under the hands of Dr. Snow himself, will be calculated to moderate the sanguine expectations of the advocates of amylene.—*Charleston Medical Journal.*

